# Putative null distributions corresponding to tests of differential expression in the Golden Spike dataset are intensity dependent

**DOI:** 10.1186/1471-2164-8-105

**Published:** 2007-04-19

**Authors:** Daniel P Gaile, Jeffrey C Miecznikowski

**Affiliations:** 1 Department of Biostatistics, University at Buffalo, Buffalo, New York, USA; 2 New York State Center of Excellence in Bioinformatics and Life Sciences, Buffalo, New York, USA

## Abstract

**Background:**

We provide a re-analysis of the Golden Spike dataset, a first generation "spike-in" control microarray dataset. The original analysis of the Golden Spike dataset was presented in a manuscript by Choe et al. and raised questions concerning the performance of several statistical methods for the control of the false discovery rate (across a set of tests for differential expression). These original findings are now in question as it has been reported that the p-values associated with the tests of differential expression for null probesets (i.e., probesets designed to be fold change 1 across the two arms of the experiment) are not uniformly distributed. Two recent publications have speculated as to the reasons the null distributions are non-uniform. A publication by Dabney and Storey concludes that the non-uniform distributions of null p-values are the direct consequence of an experimental design which requires technical replicates to approximate biological replicates. Irizarry et al. identify four characteristics of the feature level data (three related to experimental design and one artifact). Irizarry et al. argue that the four observed characteristics imply that the assumptions common to most pre-processing algorithms are not satisfied and hence the expression measure methodologies considered by Choe et al. are likely to be flawed.

**Results:**

We replicate and extend the analyses of Dabney and Storey and present our results in the context of a two stage analysis. We provide evidence that the Stage I pre-processing algorithms considered in Dabney and Storey fail to provide expression values that are adequately centered or scaled. Furthermore, we demonstrate that the distributions of the p-values, test statistics, and probabilities associated with the relative locations and variabilities of the Stage II expression values vary with signal intensity. We provide diagnostic plots and a simple logistic regression based test statistic to detect these intensity related defects in the processed data.

**Conclusion:**

We agree with Dabney and Storey that the null p-values considered in Choe et al. are indeed non-uniform. We also agree with the conclusion that, given current pre-processing technologies, the Golden Spike dataset should not serve as a reference dataset to evaluate false discovery rate controlling methodologies. However, we disagree with the assessment that the non-uniform p-values are merely the byproduct of testing for differential expression under the incorrect assumption that chip data are approximate to biological replicates. Whereas Dabney and Storey attribute the non-uniform p-values to violations of the Stage II model assumptions, we provide evidence that the non-uniformity can be attributed to the failure of the Stage I analyses to correct for systematic biases in the raw data matrix. Although we do not speculate as to the root cause of these systematic biases, the observations made in Irizarry et al. appear to be consistent with our findings. Whereas Irizarry et al. describe the effect of the experimental design on the feature level data, we consider the effect on the underlying multivariate distribution of putative null p-values. We demonstrate that the putative null distributions corresponding to the pre-processing algorithms considered in Choe et al. are all intensity dependent. This dependence serves to invalidate statistical inference based upon standard two sample test statistics. We identify a flaw in the characterization of the appropriate "null" probesets described in Choe et al. and we provide a corrected analysis which reduces (but does not eliminate) the intensity dependent effects.

## Background

Normalization of microarray data is essential for removing systematic variation and biases that are present due to the nature of the assay. In experiments where the goal is to determine differential expression scientists have developed a variety of tests and algorithms to identify differentially expressed genes. One such experiment was the "Golden Spike" experiments by [1]. In the experiment six Affymetrix chips were divided into two groups: a control group (C) and a spike group (S). The S sample contains the same cRNAs as the C sample, except for ten selected groups of approximately 130 cRNAs per group that are present at a defined increased concentration compared to the C sample. This results in 3860 cRNAs, where 1309 cRNAs are spiked in with differing concentrations between the S and C samples. The rest (2551) are present at identical relative concentration between the two sets of microarrays. This type of experiment models the general paradigm of experiments meant to detect differential expression. Recently, however, the validity of inference based upon the Golden Spike experiment has been questioned [2].

**Figure 1:**
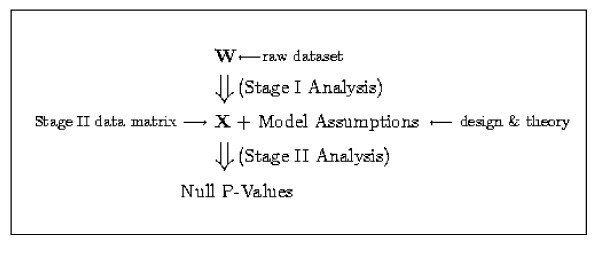
A two-stage procedure was used to obtain the sets of null p-values referenced in [1] and [2]. The first stage of the procedure involves the application of algorithms designed to correct and normalize the raw data matrix, **W**. The second stage of the procedure involves the evaluation of a test statistic for differential expression using information from the Stage II data matrix, **X**. [1] considered 150+ unique combinations of algorithms and input parameter values for the Stage I analysis and proposed a subset of the 10 best. [2] determined that the distributions of the null p-values for the [1] 10 best Stage I analyses were non-uniform for the most common choices for the Stage II analysis (e.g., t-test, permutation t-test, and Wilcoxon Rank Sum test). [2] concluded that this non-uniformity implies that the technical replicates generated by the experiment do not constitute adequate approximations of biological replicates and hence, the Stage II model assumptions associated with these tests are not met. We provide evidence that the non-uniformity can be attributed, at least in part, to the failure of the Stage I analyses to correct for systematic biases in the raw data matrix, **W**.

A key component to the Golden Spike dataset is knowledge of the null p-values for tests of differential expression, that is, information of the genes that are present in a 1:1 ratio on the S chips and the C chips provides knowledge of which tests for differential expression are truly null. Figure 1 provides a schematic of the two stage procedure used to obtain the sets of null p-values referenced in [1] and [2]. The raw Golden Spike dataset consists of data generated by the scanning device used to measure the relative spot fluorescence values across each microarray chip. For oligonucleotide (Affymetrix) experiments such as the Golden Spike, the nature of the design demands heavy statistical intervention.

In microarray experiments, the end-stage analysis usually consists of simple two-sample test statistics such as the t-statistic or the Wilcoxon Rank Sum test statistic to test for differential expression. However, it is important to note that these statistics generally operate upon data matrices which have been subjected to potentially significant amounts of pre-processing. With this technology, there are several steps required in order to process the data in order to achieve a single value representing the intensity for a given probe. It is worthwhile to consider the Affymetrix data acquisition in two stages. A Stage I analysis includes image processing where each spot is deemed to consist of a collection of pixels. From the collection of pixels at a spot an overall signal value is determined by taking a summary measure (often a median) of the pixel set at each hybridization location on the chip. In the Affymetrix data design there are 11 probe pairs spotted for each gene or SNP. Each probe pair contains two 25-mer DNA oligonucleotide probes; the perfect match (PM) probe matches perfectly to the target RNA, and the mismatch (MM) probe which is identical to its PM partner probe except for a single homomeric mismatch at the central base-pair position. The MM probe serves to estimate the nonspecific signal. In this stage, the PM and MM signals are combined into one score representing the expression signal for a specific probe. The major software packages for Stage I analysis include Bioconductor's "Affy" package, dChip and MAS 5.0 executables [1]. Each software package varies in how the image processing is performed and how the PM and MM values are combined. After obtaining a signal for each probe, the next step in the Stage I analysis is to "normalize" the data accounting for between chip effects, spatial effects, intensity effects, a possible grid effect, and any nonlinear intensity/variation effects. Popular normalizing methods include lowess and loess smoothers to remove systematic sources of noise [3,4].

The Stage I analysis often involves a matrix of dimension *p *by *m *where the *p *rows refer to the different probes, and the *m *columns refer to the different chips. The general procedure in normalizing this data is to use loess smoothers on the data set. One of the motivations for the Golden Spike experiment was to examine the numerous and varied normalization methods that currently exist for this data. Most of the normalization methods consider the data as a function of the matrix column. The goal of any of these normalization schemes is to reduce the systematic variation that exists in each chip. By considering each column of the data matrix as a separate chip, in each column we can scale and center the values, via loess smoothers so that each column has roughly the same "center" and "scale." This general approach (as discussed in [3]) does not deal well with nonlinear relationships between arrays. Another method from [3] is to transform the data via quantile regression so that the distribution of probe intensities is roughly the same across arrays. At this stage, the normalization should result in a dataset where the systematic variation is reduced in order to get a clearer glimpse of the biological variatio that is present in these experiments.

Ultimately, the Stage I analysis results in an *X *matrix of dimension *p *by *m *for each experiment where the *p *rows correspond to each (smoothed) probe value and the *m *columns correspond to the sample. In the Golden Spike datasets, numerous options in the Stage I analysis were examined, resulting in 152 different *X *matrices with each matrix corresponding to a different set of parameters in a Stage I analysis. From these 152 datasets, 10 "best" datasets were chosen that represented the best combination of processing in terms of detecting approximately 95 percent of true differentially expressed genes (DEGs) with changes greater than twofold, but less than 30 percent with changes below 1.7 fold before exceeding a 10 percent false-discovery rate. At this point, each data matrix *X *represents the input for Stage II analysis. The goal of Stage II analysis is to answer the researcher's questions of the experiment. Usually in the microarray setting this consists of a ranked list of genes determined to be differentially expressed between two groups such as treatment versus control. The methods of Stage II generally take in to account facets of the experimental design and allow the user to control for things like the false discovery rate (FDR) within a given two sample test environment. Often the validity of Stage II analyses depends upon the assumption that the Stage I analysis has provided a Stage II data matrix such that the two sample test statistic null p-values are uniformly distributed.

Dabney and Storey [2] provide a re-analysis of the Golden Spike dataset in which they consider the most common choices for the Stage II analysis (e.g., t-test, permutation t-test, and Wilcoxon Rank Sum test) and demonstrate that the null p-values for the Choe et al. 10 best datasets were non-uniform in all cases. The authors note that statistical methods to control the FDR require the assumption that the true null p-values are uniformly distributed and hence the Golden Spike data can not be utilized to assess the performance of such methods. Furthermore, Dabney and Storey conclude that the non-uniform distributions of p-values are the direct consequence of an experimental design which requires that technical replicates adequately approximate biological replicates. The authors provide simulation results which demonstrate that technical replicates analyzed as biological replicates can provide non-uniform null p-value distributions but fail to provide any evidence that the parameter values that evoke this behavior are consistent with the set of conditions under which the Golden Spike experiment was conducted. Presumably the reader is left to infer that because the null p-values are non-uniform and because technical replicates analyzed as biological replicates can provide non-uniform null distributions, then the technical replicates generated by the Golden Spike experiment do not adequately approximate biological replicates.

We have replicated and extended the analyses of Dabney and Storey and we agree with the assessment that the null p-values are indeed non-uniform. We also agree with the conclusion that, given current pre-processing (i.e., Stage I) technologies, the Golden Spike datasets should not serve as reference datasets to evaluate FDR controlling methodologies. However, we disagree with the assessment that the non-uniform p-values are merely the byproduct of testing for differential expression under the assumption that chip data are approximate to biological replicates when, in fact, they are not. Whereas Dabney and Storey attribute the non-uniform p-values to violations of the Stage II model assumptions, we provide evidence that the non-uniformity can be attributed to the failure of the Stage I analyses to correct for systematic biases in the raw data matrix.

A recent article by Irizarry et al. [5] identifies four characteristics of the feature level data (three related to experimental design and one artifact) which offer a possible explanation for the inconsistencies between the conclusions presented in [1] and [6]. Irizarry et al. argue that the four observed characteristics imply that the assumptions common to most pre-processing algorithms are not satisfied and hence the expression measure methodologies considered in [1] are likely to be flawed. Whereas Irizarry et al. describe the effect of the experimental design on the feature level data, we consider the effect on the underlying multivariate distribution of putative null p-values. Specifically, we demonstrate that the 10 best Stage I analyses considered in [1] and [2] provide Stage II data matrices in which the columns are neither adequately centered nor adequately scaled. Further we note that the observed deviations in centering and scaling are intensity dependent. The intensity dependence of the Stage II data values leads to putative null distributions which are intensity dependent and hence non-uniform. We provide simple diagnostic plots which indicate that the relative center and scales of the underlying distributions for the control and spike-in expression values vary as a function of signal intensity.

Although the scope of this manuscript is in large part restricted to the re-analysis of the Golden Spike dataset, we also apply several of the same diagnostic plots to another Affymetrix spike-in experiment. The results suggest that some of the intensity dependent effects may exist in other settings. We relegate the extensive application and the continued development of such diagnostics to future research.

## Results and Discussion

### Re-analysis of the Golden Spike Dataset

**Figure 2:**
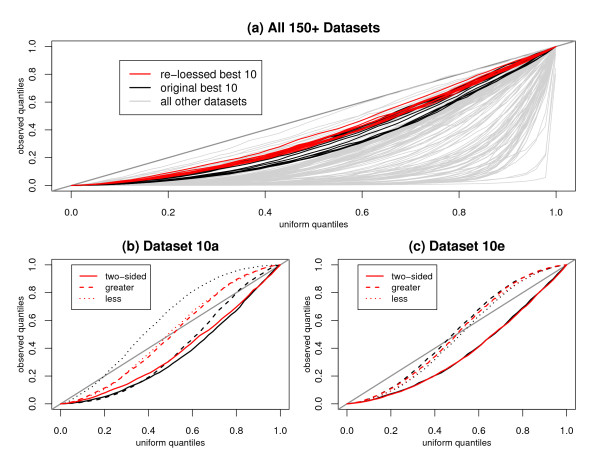
Sample quantile plots for various sets of null p- values. The x-axes correspond to the expected quantiles for a uniform distribution and the y-axes correspond to the observed (sample) quantiles. (a) Sample quantile plots for the t-test p-values associated with the 152 preprocessing combinations described by [1]. Black lines correspond to the 10 best datasets and are consistent with the curves presented in Figure 1 of [2]. The red lines correspond to re-loessed datasets that were obtained using the same combinations of preprocessing steps as the original 10 sets with the exception that the invariant subsets consisted only of the "present null" (present with fold change = 1) probesets (versus both the "present null" and "empty null" probesets used in [1]). The distribution of the p-values thus depends upon the choice of the invariant subset. (b) Sample quantile curves for dataset 10a. Solid lines correspond to the two-sided p-values and the dashed and dotted lines correspond to the p-values associated with the one sided tests. The model presented in [2] does not account for the discrepancy in the one-sided p-values observed for this dataset, which is not manifest in the re-loessed data (red lines). Similar results are seen with datasets 10b, c, d and 9a, b, c, d. (c) As in (b) but showing sample quantile curves for dataset 10e; dataset 9e is similar. The p-value discrepancies are much less pronounced for these two datasets. This figure appears with permission in the response to [2].

Our analysis of the Golden Spike Dataset reveals that the null two sample t-test p-value distributions are non-uniform across the 152 combinations of Stage I analyses. The fact that all distributions were non-uniform implies that this problem can not be attributed to the procedure utilized to identify the ten best datasets. The two sample test was conducted using the equal variance t-test so that the analysis would be consistent with the one presented in [2]. Other test statistics (i.e., Wilcoxon Rank Sum, permutation t-test, and Welch's t-test) were considered and yielded similar results, a finding which is also consistent with those reported in [2]. Figure 2 contains sample quantile plots for the 152 sets of null p-values corresponding to the 152 datasets described in [1]. The black curves in Figure 2(a) correspond to the ten best datasets (i.e., datasets labeled 9a-e and 10a-e). The grey curves in Figure 2(a) correspond to the remaining 142 datasets and demonstrate that non-uniform null p-values were observed in datasets other than the ten best.

#### Observed p-value Distributions Inconsistent with Model of Dabney and Storey

Dabney and Storey attributed the non uniform distribution of p-values to the fact that the Golden Spike experimental design requires technical replicates to masquerade as biological replicates. In their response to [2], the authors of [1] acknowledged that the three spike-in and three control chips were technical replicates but they argued that the differences in the relative concentrations of the fold change one genes within the master spike-in sample (i.e., prior to splitting into three samples) compared to those in the master control sample should have had a negligible impact on the observed expression values.

Dabney and Storey proposed the following model for *i *genes, *i *= 1, 2,...*m*, *j *treatments, *j *= *C*,*S *and *k *technical replicates, *k *= 1, 2, 3 and the Stage II expression data matrix *X*:

*X*_*ijk *_= *μ*_*ij *_+ *ε*_*ij *_+ *φ*_*ijk*_

where

[μiCμiS]=[mean of gene i for the control setmean of gene i for the spike-in set]
 MathType@MTEF@5@5@+=feaafiart1ev1aaatCvAUfKttLearuWrP9MDH5MBPbIqV92AaeXatLxBI9gBaebbnrfifHhDYfgasaacH8akY=wiFfYdH8Gipec8Eeeu0xXdbba9frFj0=OqFfea0dXdd9vqai=hGuQ8kuc9pgc9s8qqaq=dirpe0xb9q8qiLsFr0=vr0=vr0dc8meaabaqaciaacaGaaeqabaqabeGadaaakeaadaWadaqaauaabeqaceaaaeaaiiGacqWF8oqBdaWgaaWcbaGaemyAaKMaem4qameabeaaaOqaaiab=X7aTnaaBaaaleaacqWGPbqAcqWGtbWuaeqaaaaaaOGaay5waiaaw2faaiabg2da9maadmaabaqbaeaabiqaaaqaaiabb2gaTjabbwgaLjabbggaHjabb6gaUjabbccaGiabb+gaVjabbAgaMjabbccaGiabbEgaNjabbwgaLjabb6gaUjabbwgaLjabbccaGiabdMgaPjabbccaGiabbAgaMjabb+gaVjabbkhaYjabbccaGiabbsha0jabbIgaOjabbwgaLjabbccaGiabbogaJjabb+gaVjabb6gaUjabbsha0jabbkhaYjabb+gaVjabbYgaSjabbccaGiabbohaZjabbwgaLjabbsha0bqaaiabb2gaTjabbwgaLjabbggaHjabb6gaUjabbccaGiabb+gaVjabbAgaMjabbccaGiabbEgaNjabbwgaLjabb6gaUjabbwgaLjabbccaGiabdMgaPjabbccaGiabbAgaMjabb+gaVjabbkhaYjabbccaGiabbsha0jabbIgaOjabbwgaLjabbccaGiabbohaZjabbchaWjabbMgaPjabbUgaRjabbwgaLjabb2caTiabbMgaPjabb6gaUjabbccaGiabbohaZjabbwgaLjabbsha0baaaiaawUfacaGLDbaaaaa@8FEE@

[εiCεiS]~N2((00),σi2[1ρρ1])
 MathType@MTEF@5@5@+=feaafiart1ev1aaatCvAUfKttLearuWrP9MDH5MBPbIqV92AaeXatLxBI9gBaebbnrfifHhDYfgasaacH8akY=wiFfYdH8Gipec8Eeeu0xXdbba9frFj0=OqFfea0dXdd9vqai=hGuQ8kuc9pgc9s8qqaq=dirpe0xb9q8qiLsFr0=vr0=vr0dc8meaabaqaciaacaGaaeqabaqabeGadaaakeaadaWadaqaauaabeqaceaaaeaaiiGacqWF1oqzdaWgaaWcbaGaemyAaKMaem4qameabeaaaOqaaiab=v7aLnaaBaaaleaacqWGPbqAcqWGtbWuaeqaaaaaaOGaay5waiaaw2faaiabc6ha+jabd6eaonaaBaaaleaacqaIYaGmaeqaaOWaaeWaaeaadaqadaqaauaabeqaceaaaeaacqaIWaamaeaacqaIWaamaaaacaGLOaGaayzkaaGaeiilaWIae83Wdm3aa0baaSqaaiabdMgaPbqaaiabikdaYaaakmaadmaabaqbaeqabiGaaaqaaiabigdaXaqaaiab=f8aYbqaaiab=f8aYbqaaiabigdaXaaaaiaawUfacaGLDbaaaiaawIcacaGLPaaaaaa@4CA1@

and *φ*_*ijk*_*~N *(0, τi2
 MathType@MTEF@5@5@+=feaafiart1ev1aaatCvAUfKttLearuWrP9MDH5MBPbIqV92AaeXatLxBI9gBaebbnrfifHhDYfgasaacH8akY=wiFfYdH8Gipec8Eeeu0xXdbba9frFj0=OqFfea0dXdd9vqai=hGuQ8kuc9pgc9s8qqaq=dirpe0xb9q8qiLsFr0=vr0=vr0dc8meaabaqaciaacaGaaeqabaqabeGadaaakeaaiiGacqWFepaDdaqhaaWcbaGaemyAaKgabaGaeGOmaidaaaaa@30F2@). In the model stated in Equation (1), straightforward calculations show that for gene *i *we have the following distribution:

[xiC1xiC2xiC3xiS1xiS2xiS3]~N6((000000), [σi2+τi2σi2σi2σi2σi2+τi2σi2σi2σi2σi2+τi2σi2ρσi2ρσi2ρσi2ρσi2ρσi2ρσi2ρσi2ρσi2ρσi2ρσi2ρσi2ρσi2ρσi2ρσi2ρσi2ρσi2ρσi2ρσi2+τi2σi2σi2σi2σi2+τi2σi2σi2σi2σi2+τi2])
 MathType@MTEF@5@5@+=feaafiart1ev1aaatCvAUfKttLearuWrP9MDH5MBPbIqV92AaeXatLxBI9gBaebbnrfifHhDYfgasaacH8akY=wiFfYdH8Gipec8Eeeu0xXdbba9frFj0=OqFfea0dXdd9vqai=hGuQ8kuc9pgc9s8qqaq=dirpe0xb9q8qiLsFr0=vr0=vr0dc8meaabaqaciaacaGaaeqabaqabeGadaaakeaadaWadaqaauaabeqageaaaaqaaiabdIha4naaBaaaleaacqWGPbqAcqWGdbWqcqaIXaqmaeqaaaGcbaGaemiEaG3aaSbaaSqaaiabdMgaPjabdoeadjabikdaYaqabaaakeaacqWG4baEdaWgaaWcbaGaemyAaKMaem4qamKaeG4mamdabeaaaOqaaiabdIha4naaBaaaleaacqWGPbqAcqWGtbWucqaIXaqmaeqaaaGcbaGaemiEaG3aaSbaaSqaaiabdMgaPjabdofatjabikdaYaqabaaakeaacqWG4baEdaWgaaWcbaGaemyAaKMaem4uamLaeG4mamdabeaaaaaakiaawUfacaGLDbaacqGG+bGFcqWGobGtdaWgaaWcbaGaeGOnaydabeaakmaabmaabaWaaeWaaeaafaqabeGbbaaaaeaacqaIWaamaeaacqaIWaamaeaacqaIWaamaeaacqaIWaamaeaacqaIWaamaeaacqaIWaamaaaacaGLOaGaayzkaaGaeiilaWIaeeiiaaYaamWaaeaafaqabeGacqabbaqbaeqabmWaaaqaaGGaciab=n8aZnaaDaaaleaacqWGPbqAaeaacqaIYaGmaaGccqGHRaWkcqWFepaDdaqhaaWcbaGaemyAaKgabaGaeGOmaidaaaGcbaGae83Wdm3aa0baaSqaaiabdMgaPbqaaiabikdaYaaaaOqaaiab=n8aZnaaDaaaleaacqWGPbqAaeaacqaIYaGmaaaakeaacqWFdpWCdaqhaaWcbaGaemyAaKgabaGaeGOmaidaaaGcbaGae83Wdm3aa0baaSqaaiabdMgaPbqaaiabikdaYaaakiabgUcaRiab=r8a0naaDaaaleaacqWGPbqAaeaacqaIYaGmaaaakeaacqWFdpWCdaqhaaWcbaGaemyAaKgabaGaeGOmaidaaaGcbaGae83Wdm3aa0baaSqaaiabdMgaPbqaaiabikdaYaaaaOqaaiab=n8aZnaaDaaaleaacqWGPbqAaeaacqaIYaGmaaaakeaacqWFdpWCdaqhaaWcbaGaemyAaKgabaGaeGOmaidaaOGaey4kaSIae8hXdq3aa0baaSqaaiabdMgaPbqaaiabikdaYaaaaaaakeaafaqabeWadaaabaGae83Wdm3aa0baaSqaaiabdMgaPbqaaiabikdaYaaakiab=f8aYbqaaiab=n8aZnaaDaaaleaacqWGPbqAaeaacqaIYaGmaaGccqWFbpGCaeaacqWFdpWCdaqhaaWcbaGaemyAaKgabaGaeGOmaidaaOGae8xWdihabaGae83Wdm3aa0baaSqaaiabdMgaPbqaaiabikdaYaaakiab=f8aYbqaaiab=n8aZnaaDaaaleaacqWGPbqAaeaacqaIYaGmaaGccqWFbpGCaeaacqWFdpWCdaqhaaWcbaGaemyAaKgabaGaeGOmaidaaOGae8xWdihabaGae83Wdm3aa0baaSqaaiabdMgaPbqaaiabikdaYaaakiab=f8aYbqaaiab=n8aZnaaDaaaleaacqWGPbqAaeaacqaIYaGmaaGccqWFbpGCaeaacqWFdpWCdaqhaaWcbaGaemyAaKgabaGaeGOmaidaaOGae8xWdihaaaqaauaabeqadmaaaeaacqWFdpWCdaqhaaWcbaGaemyAaKgabaGaeGOmaidaaOGae8xWdihabaGae83Wdm3aa0baaSqaaiabdMgaPbqaaiabikdaYaaakiab=f8aYbqaaiab=n8aZnaaDaaaleaacqWGPbqAaeaacqaIYaGmaaGccqWFbpGCaeaacqWFdpWCdaqhaaWcbaGaemyAaKgabaGaeGOmaidaaOGae8xWdihabaGae83Wdm3aa0baaSqaaiabdMgaPbqaaiabikdaYaaakiab=f8aYbqaaiab=n8aZnaaDaaaleaacqWGPbqAaeaacqaIYaGmaaGccqWFbpGCaeaacqWFdpWCdaqhaaWcbaGaemyAaKgabaGaeGOmaidaaOGae8xWdihabaGae83Wdm3aa0baaSqaaiabdMgaPbqaaiabikdaYaaakiab=f8aYbqaaiab=n8aZnaaDaaaleaacqWGPbqAaeaacqaIYaGmaaGccqWFbpGCaaaabaqbaeqabmWaaaqaaiab=n8aZnaaDaaaleaacqWGPbqAaeaacqaIYaGmaaGccqGHRaWkcqWFepaDdaqhaaWcbaGaemyAaKgabaGaeGOmaidaaaGcbaGae83Wdm3aa0baaSqaaiabdMgaPbqaaiabikdaYaaaaOqaaiab=n8aZnaaDaaaleaacqWGPbqAaeaacqaIYaGmaaaakeaacqWFdpWCdaqhaaWcbaGaemyAaKgabaGaeGOmaidaaaGcbaGae83Wdm3aa0baaSqaaiabdMgaPbqaaiabikdaYaaakiabgUcaRiab=r8a0naaDaaaleaacqWGPbqAaeaacqaIYaGmaaaakeaacqWFdpWCdaqhaaWcbaGaemyAaKgabaGaeGOmaidaaaGcbaGae83Wdm3aa0baaSqaaiabdMgaPbqaaiabikdaYaaaaOqaaiab=n8aZnaaDaaaleaacqWGPbqAaeaacqaIYaGmaaaakeaacqWFdpWCdaqhaaWcbaGaemyAaKgabaGaeGOmaidaaOGaey4kaSIae8hXdq3aa0baaSqaaiabdMgaPbqaaiabikdaYaaaaaaaaaGccaGLBbGaayzxaaaacaGLOaGaayzkaaaaaa@3554@

If we consider the linear combination:

Wi=X¯iS−X¯iC
 MathType@MTEF@5@5@+=feaafiart1ev1aaatCvAUfKttLearuWrP9MDH5MBPbIqV92AaeXatLxBI9gBaebbnrfifHhDYfgasaacH8akY=wiFfYdH8Gipec8Eeeu0xXdbba9frFj0=OqFfea0dXdd9vqai=hGuQ8kuc9pgc9s8qqaq=dirpe0xb9q8qiLsFr0=vr0=vr0dc8meaabaqaciaacaGaaeqabaqabeGadaaakeaacqWGxbWvdaWgaaWcbaGaemyAaKgabeaakiabg2da9iqbdIfayzaaraWaaSbaaSqaaiabdMgaPjabdofatbqabaGccqGHsislcuWGybawgaqeamaaBaaaleaacqWGPbqAcqWGdbWqaeqaaaaa@395F@

where X¯
 MathType@MTEF@5@5@+=feaafiart1ev1aaatCvAUfKttLearuWrP9MDH5MBPbIqV92AaeXatLxBI9gBaebbnrfifHhDYfgasaacH8akY=wiFfYdH8Gipec8Eeeu0xXdbba9frFj0=OqFfea0dXdd9vqai=hGuQ8kuc9pgc9s8qqaq=dirpe0xb9q8qiLsFr0=vr0=vr0dc8meaabaqaciaacaGaaeqabaqabeGadaaakeaacuWGybawgaqeaaaa@2DFD@_*iS*_, and X¯
 MathType@MTEF@5@5@+=feaafiart1ev1aaatCvAUfKttLearuWrP9MDH5MBPbIqV92AaeXatLxBI9gBaebbnrfifHhDYfgasaacH8akY=wiFfYdH8Gipec8Eeeu0xXdbba9frFj0=OqFfea0dXdd9vqai=hGuQ8kuc9pgc9s8qqaq=dirpe0xb9q8qiLsFr0=vr0=vr0dc8meaabaqaciaacaGaaeqabaqabeGadaaakeaacuWGybawgaqeaaaa@2DFD@_*iC *_represent the sample mean for probe *i *under condition *S *and *C*, respectively, then it follows that

Wi~N(μS−μC,2(1−ρ)σi2+23τi2).
 MathType@MTEF@5@5@+=feaafiart1ev1aaatCvAUfKttLearuWrP9MDH5MBPbIqV92AaeXatLxBI9gBaebbnrfifHhDYfgasaacH8akY=wiFfYdH8Gipec8Eeeu0xXdbba9frFj0=OqFfea0dXdd9vqai=hGuQ8kuc9pgc9s8qqaq=dirpe0xb9q8qiLsFr0=vr0=vr0dc8meaabaqaciaacaGaaeqabaqabeGadaaakeaacqWGxbWvdaWgaaWcbaGaemyAaKgabeaakiabc6ha+jabd6eaojabcIcaOGGaciab=X7aTnaaBaaaleaacqWGtbWuaeqaaOGaeyOeI0Iae8hVd02aaSbaaSqaaiabdoeadbqabaGccqGGSaalcqaIYaGmcqGGOaakcqaIXaqmcqGHsislcqWFbpGCcqGGPaqkcqWFdpWCdaqhaaWcbaGaemyAaKgabaGaeGOmaidaaOGaey4kaSYaaSaaaeaacqaIYaGmaeaacqaIZaWmaaGae8hXdq3aa0baaSqaaiabdMgaPbqaaiabikdaYaaakiabcMcaPiabc6caUaaa@4E32@

The standard two-sample t-statistic is given by

T=X¯iS−X¯iCsS2/3+sC2/3.
 MathType@MTEF@5@5@+=feaafiart1ev1aaatCvAUfKttLearuWrP9MDH5MBPbIqV92AaeXatLxBI9gBaebbnrfifHhDYfgasaacH8akY=wiFfYdH8Gipec8Eeeu0xXdbba9frFj0=OqFfea0dXdd9vqai=hGuQ8kuc9pgc9s8qqaq=dirpe0xb9q8qiLsFr0=vr0=vr0dc8meaabaqaciaacaGaaeqabaqabeGadaaakeaacqWGubavcqGH9aqpdaWcaaqaaiqbdIfayzaaraWaaSbaaSqaaiabdMgaPjabdofatbqabaGccqGHsislcuWGybawgaqeamaaBaaaleaacqWGPbqAcqWGdbWqaeqaaaGcbaWaaOaaaeaacqWGZbWCdaqhaaWcbaGaem4uamfabaGaeGOmaidaaOGaei4la8IaeG4mamJaey4kaSIaem4Cam3aa0baaSqaaiabdoeadbqaaiabikdaYaaakiabc+caViabiodaZaWcbeaaaaGccqGGUaGlaaa@44EF@

where *s*_*S *_and *s*_*C *_represent the sample standard deviation of probe *i *under condition *S *and *C *respectively. It follows from (3) that a t-test statistic calculated with respect to random variables governed by model (1) constitutes an observation from a distribution which is heavier in the tails than a *t*_4 _distribution provided 2(1 - *ρ*)σi2
 MathType@MTEF@5@5@+=feaafiart1ev1aaatCvAUfKttLearuWrP9MDH5MBPbIqV92AaeXatLxBI9gBaebbnrfifHhDYfgasaacH8akY=wiFfYdH8Gipec8Eeeu0xXdbba9frFj0=OqFfea0dXdd9vqai=hGuQ8kuc9pgc9s8qqaq=dirpe0xb9q8qiLsFr0=vr0=vr0dc8meaabaqaciaacaGaaeqabaqabeGadaaakeaaiiGacqWFdpWCdaqhaaWcbaGaemyAaKgabaGaeGOmaidaaaaa@30F0@*> *0. This follows from the fact that square of the denominator of the test statistic is an unbiased estimator of 23τi2
 MathType@MTEF@5@5@+=feaafiart1ev1aaatCvAUfKttLearuWrP9MDH5MBPbIqV92AaeXatLxBI9gBaebbnrfifHhDYfgasaacH8akY=wiFfYdH8Gipec8Eeeu0xXdbba9frFj0=OqFfea0dXdd9vqai=hGuQ8kuc9pgc9s8qqaq=dirpe0xb9q8qiLsFr0=vr0=vr0dc8meaabaqaciaacaGaaeqabaqabeGadaaakeaadaWcaaqaaiabikdaYaqaaiabiodaZaaaiiGacqWFepaDdaqhaaWcbaGaemyAaKgabaGaeGOmaidaaaaa@32E8@ and hence, a negatively biased estimator of the variance of *W*_*i*_. Hence, evaluating t-test statistics such as (4) against a *t*_4 _distribution will provide p-values which are negatively biased. This is the crux of the Dabney and Storey critique of the Golden Spike experimental design. Unfortunately the experimental design does not provide enough data to fit model (1) and directly estimate the relative magnitudes of σi2
 MathType@MTEF@5@5@+=feaafiart1ev1aaatCvAUfKttLearuWrP9MDH5MBPbIqV92AaeXatLxBI9gBaebbnrfifHhDYfgasaacH8akY=wiFfYdH8Gipec8Eeeu0xXdbba9frFj0=OqFfea0dXdd9vqai=hGuQ8kuc9pgc9s8qqaq=dirpe0xb9q8qiLsFr0=vr0=vr0dc8meaabaqaciaacaGaaeqabaqabeGadaaakeaaiiGacqWFdpWCdaqhaaWcbaGaemyAaKgabaGaeGOmaidaaaaa@30F0@ and τi2
 MathType@MTEF@5@5@+=feaafiart1ev1aaatCvAUfKttLearuWrP9MDH5MBPbIqV92AaeXatLxBI9gBaebbnrfifHhDYfgasaacH8akY=wiFfYdH8Gipec8Eeeu0xXdbba9frFj0=OqFfea0dXdd9vqai=hGuQ8kuc9pgc9s8qqaq=dirpe0xb9q8qiLsFr0=vr0=vr0dc8meaabaqaciaacaGaaeqabaqabeGadaaakeaaiiGacqWFepaDdaqhaaWcbaGaemyAaKgabaGaeGOmaidaaaaa@30F2@. However, it is still possible to determine that model (1) does not adequately explain all aspects of the observed p-value distributions for all Stage II datasets. There is an underlying symmetry to this putative model mis-specification because if the t-test statistic underestimates the actual variance, then the distributions of the one-sided p-values should be parsimonious with the distribution of the two-sided p-values. In actuality, the one-sided p-values for eight of the ten best datasets proved to be inconsistent with the two sided p-values. Figure 2(b) contains the sample quantile curves for dataset 10a where solid lines correspond to the two-sided p-values and the dashed and dotted lines correspond to the p-values associated with the one sided tests. The distributions of the one-sided p-values are not in agreement. Surprisingly, the set of p-values associated with the "less than" alternative appearing to contain a disproportionate number of large p-values and an insufficient number of small p-values. Datasets 9a-d and 10b-d provided results similar to those observed for dataset 10a. Figure 2(c) contains the sample quantile curves for dataset 10e in which the two sets of one-sided p-values appear to share the same underlying distribution.

**Figure 3:**
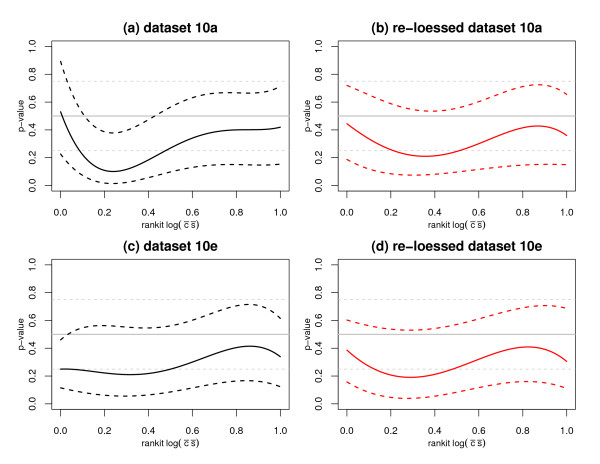
Estimates of the null p-value quartiles vary as a function of signal intensity for datasets 10a (a, b) and 10e (c, d); although less so for the re-loessed data. The x-axes correspond to the rankit (i.e., rank of valuetotal#of values+1
 MathType@MTEF@5@5@+=feaafiart1ev1aaatCvAUfKttLearuWrP9MDH5MBPbIqV92AaeXatLxBI9gBaebbnrfifHhDYfgasaacH8akY=wiFfYdH8Gipec8Eeeu0xXdbba9frFj0=OqFfea0dXdd9vqai=hGuQ8kuc9pgc9s8qqaq=dirpe0xb9q8qiLsFr0=vr0=vr0dc8meaabaqaciaacaGaaeqabaqabeGadaaakeaadaWcaaqaaiabbkhaYjabbggaHjabb6gaUjabbUgaRjabbccaGiabb+gaVjabbAgaMjabbccaGiabbAha2jabbggaHjabbYgaSjabbwha1jabbwgaLbqaaiabbsha0jabb+gaVjabbsha0jabbggaHjabbYgaSjabcocaJiabb+gaVjabbAgaMjabbccaGiabbAha2jabbggaHjabbYgaSjabbwha1jabbwgaLjabbohaZjabgUcaRiabigdaXaaaaaa@52A7@) of the log of the product of the expression means. The y-axes correspond the observed two-sided p-values. Solid and dashed grey lines indicate the theoretical medians and quartiles, respectively. The null p-values were modeled as a function of a 4^*th *^order polynomial for rankit intensity. Black and red lines correspond to the quantile regression fits for *τ *= 0.5 (solid) and *τ *= 0.25, 0.75 (dashed). Portions of this figure appear with permission in the response to [2].

**Figure 4:**
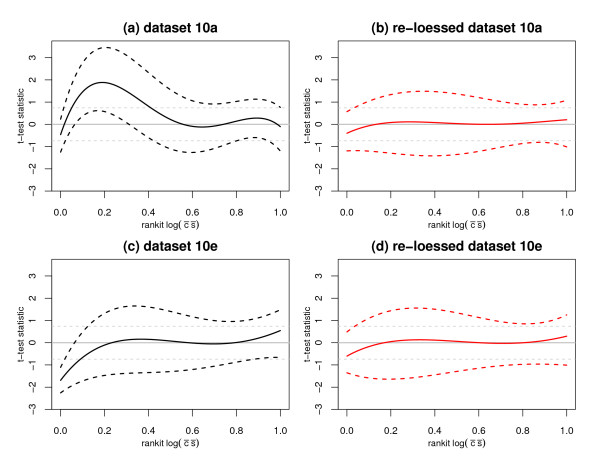
Estimates of the null t-test statistic quartiles vary as a function of signal intensity for datasets 10a (a, b) and 10e (c, d); although less so for the re-loessed data. The x-axes correspond to the rankit (i.e., rank of valuetotal#of values+1
 MathType@MTEF@5@5@+=feaafiart1ev1aaatCvAUfKttLearuWrP9MDH5MBPbIqV92AaeXatLxBI9gBaebbnrfifHhDYfgasaacH8akY=wiFfYdH8Gipec8Eeeu0xXdbba9frFj0=OqFfea0dXdd9vqai=hGuQ8kuc9pgc9s8qqaq=dirpe0xb9q8qiLsFr0=vr0=vr0dc8meaabaqaciaacaGaaeqabaqabeGadaaakeaadaWcaaqaaiabbkhaYjabbggaHjabb6gaUjabbUgaRjabbccaGiabb+gaVjabbAgaMjabbccaGiabbAha2jabbggaHjabbYgaSjabbwha1jabbwgaLbqaaiabbsha0jabb+gaVjabbsha0jabbggaHjabbYgaSjabcocaJiabb+gaVjabbAgaMjabbccaGiabbAha2jabbggaHjabbYgaSjabbwha1jabbwgaLjabbohaZjabgUcaRiabigdaXaaaaaa@52A7@) of the log of the product of the expression means. The y-axes correspond the observed two-sided t-test statistics. Solid and dashed grey lines indicate the theoretical medians and quartiles, respectively. The null t-test statistics were modeled as a function of a 4^*th *^order polynomial for rankit intensity. Black and red lines correspond to the quantile regression fits for *τ *= 0.5 (solid) and *τ *= 0.25, 0.75 (dashed). The overwhelming positive deviation of the null distribution in (a) is consistent with the discrepancy between the one-sided p-values observed in Figure 2(b). Portions of this figure appear with permission in the response to [2].

Most importantly, the model (1) does not adequately explain the most intriguing aspect of the observed p-value distributions for all Stage II datasets; that the distributions are not invariant with respect to the overall signal intensity. Figures 3(a) and 3(c) contain curves which estimate the underlying population quartiles for the p-value distributions as a function of signal intensity for datasets 10a and 10e. The observed p-values were modeled as a function of a 4^*th *^order polynomial for rankit intensity, rank of valuetotal#of values+1
 MathType@MTEF@5@5@+=feaafiart1ev1aaatCvAUfKttLearuWrP9MDH5MBPbIqV92AaeXatLxBI9gBaebbnrfifHhDYfgasaacH8akY=wiFfYdH8Gipec8Eeeu0xXdbba9frFj0=OqFfea0dXdd9vqai=hGuQ8kuc9pgc9s8qqaq=dirpe0xb9q8qiLsFr0=vr0=vr0dc8meaabaqaciaacaGaaeqabaqabeGadaaakeaadaWcaaqaaiabbkhaYjabbggaHjabb6gaUjabbUgaRjabbccaGiabb+gaVjabbAgaMjabbccaGiabbAha2jabbggaHjabbYgaSjabbwha1jabbwgaLbqaaiabbsha0jabb+gaVjabbsha0jabbggaHjabbYgaSjabcocaJiabb+gaVjabbAgaMjabbccaGiabbAha2jabbggaHjabbYgaSjabbwha1jabbwgaLjabbohaZjabgUcaRiabigdaXaaaaaa@52A7@. The curves were fit using quantile regression [7,8] where the black lines correspond to the fits for *τ *= 0.5 (solid) and *τ *= 0.25, 0.75 (dashed). Solid and dashed grey lines indicate the theoretical medians and quartiles, respectively. Inspection of Figure 3(a) reveals that the p-values for dataset 10a are negatively biased across all intensities but that the magnitude of the bias is intensity variant. Inspection of Figure 3(b) reveals that the p-values for dataset 10e are also negatively biased across all intensities but that the magnitude of the bias does not vary with intensity to the extent which was observed for dataset 10a. Figures 4(a) and 4(c) contain curves which estimate the underlying population quartiles for the t-test distributions as a function of signal intensity for datasets 10a and 10e. As in the previous figure, the observed t-tests were modeled as a function of a 4^*th *^order polynomial for rankit intensity. The curves were fit using the quantile regression and are coded as in Figures 3(a) and 3(c). For dataset 10a, the test statistics corresponding to the null genes with overall signal intensities falling below the median all appear to be positively biased and exhibit a greater degree of variation than is compatible with the null *t*_4 _distribution. This observation is consistent with the previous observation that the p-values associated with the "less than" alternative contain an excessive number of large p-values. For dataset 10e, the test statistics corresponding the null genes with overall signal intensities falling in the lower 15–20% appear to be negatively biased while test statistics corresponding the null genes with overall signal intensities falling in the upper 15% appear to be positively biased. The results in Figures 2(c) and 4(c) suggest that the effect that these biases have upon the relative distributions of the one-sided p-values appears to wash out across all signal intensities even though the distributions are different for genes with overall signal intensities at the extremes.

#### Re-Loessing Golden Spike Dataset Improves Null Distributions

Each of the ten best Choe et al. Stage II data matrices were obtained using Stage I steps that included correcting the observed intensity with a loess curve that was fit to values from an invariant set of genes. This invariant set included present null (i.e., present with a putative fold change of one) as well as empty null (i.e., not present in either sample) probesets. The inclusion of the empty null probesets appears to have had a deleterious effect on the distributions of the null p-values. We have calculated a new set of ten best datasets in which the invariant set contains only the present null probesets. These calculations were performed at our request by the authors of [1] using analysis scripts which were identical to those used for the original analyses except for passages of the code relating to the identification of the invariant set. The red curves in Figures 2(a)–(c) correspond to the sample quantile curves for the re-loessed datasets. The "re-loessed" datasets are still significantly non-uniform, although noticeably less so than the original datasets. Inclusion of the empty nulls in the original invariant sets appears to have contributed to the observed biases in the underlying t-distributions as inspection of Figures 4(b) and 4(d) indicates that this bias appears to be mitigated in the re-loessed datasets.

#### Other Common Two Sample Tests Failed to Provide Uniform Null Distributions

**Figure 5:**
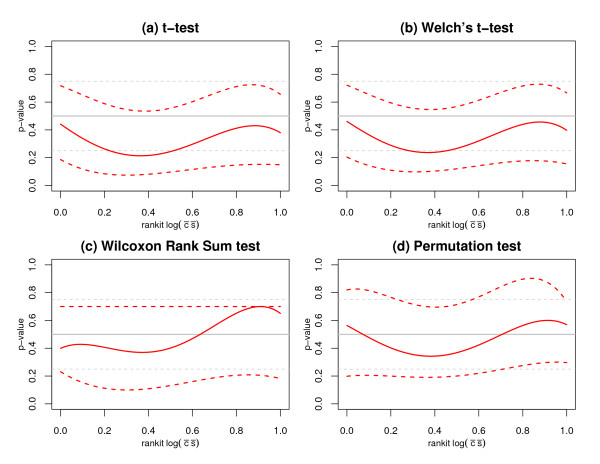
Estimates of the null p-value quartiles vary as a function of signal intensity for four common two sample test procedures applied to the re-loessed dataset 10a. The x-axes correspond to the rankit (i.e., rank of valuetotal#of values+1
 MathType@MTEF@5@5@+=feaafiart1ev1aaatCvAUfKttLearuWrP9MDH5MBPbIqV92AaeXatLxBI9gBaebbnrfifHhDYfgasaacH8akY=wiFfYdH8Gipec8Eeeu0xXdbba9frFj0=OqFfea0dXdd9vqai=hGuQ8kuc9pgc9s8qqaq=dirpe0xb9q8qiLsFr0=vr0=vr0dc8meaabaqaciaacaGaaeqabaqabeGadaaakeaadaWcaaqaaiabbkhaYjabbggaHjabb6gaUjabbUgaRjabbccaGiabb+gaVjabbAgaMjabbccaGiabbAha2jabbggaHjabbYgaSjabbwha1jabbwgaLbqaaiabbsha0jabb+gaVjabbsha0jabbggaHjabbYgaSjabcocaJiabb+gaVjabbAgaMjabbccaGiabbAha2jabbggaHjabbYgaSjabbwha1jabbwgaLjabbohaZjabgUcaRiabigdaXaaaaaa@52A7@) of the log of the product of the expression means. The y-axes correspond the observed two-sided p-values. Solid and dashed grey lines indicate the theoretical medians and quartiles, respectively. The null p-values were modeled as a function of a 4^*th *^order polynomial for rankit intensity. Red lines correspond to the quantile regression fits for *τ *= 0.5 (solid) and *τ *= 0.25, 0.75 (dashed). (a) P-values for the two sample t-test conducted under the assumption of equal variances. (b) P-values for the two sample t-test conducted under the assumption of unequal variances (a.k.a. Welch's test). Relaxing the assumption of equal variances provides for only a slight improvement in the distribution of the null p-values (c) P-values for the Wilcoxon Rank Sum test. (d) P-values for the permutation test [19,20]. Permutation based approaches are not robust to the systematic errors manifest in the Stage II data matrix.

Although removal of the empty nulls from the invariant set provides data that is better centered than the original ten best, the results depicted in Figures 3(b) and 3(d) indicate that the p-value distributions are still non-uniform and intensity dependent. We re-analyzed the re-loessed ten best datasets using three other common two sample test procedures and found that none were robust to the problems which remain in the underlying Stage II data matrices. Figure 5 contains the results of this analysis for re-loessed dataset 10a.

### Distribution Free Diagnostic Plots

**Figure 6:**
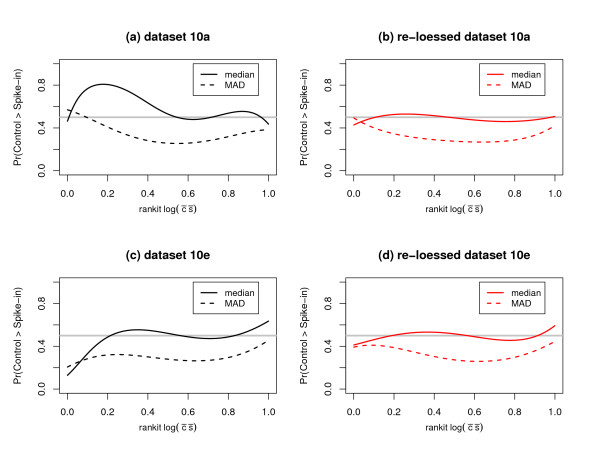
A diagnostic plot to assess, as a function of signal intensity, whether or not the underlying distributions for the control and spike-in expression values share the same center and scale. The x-axes correspond to the rankit (i.e., rank of valuetotal#of values+1
 MathType@MTEF@5@5@+=feaafiart1ev1aaatCvAUfKttLearuWrP9MDH5MBPbIqV92AaeXatLxBI9gBaebbnrfifHhDYfgasaacH8akY=wiFfYdH8Gipec8Eeeu0xXdbba9frFj0=OqFfea0dXdd9vqai=hGuQ8kuc9pgc9s8qqaq=dirpe0xb9q8qiLsFr0=vr0=vr0dc8meaabaqaciaacaGaaeqabaqabeGadaaakeaadaWcaaqaaiabbkhaYjabbggaHjabb6gaUjabbUgaRjabbccaGiabb+gaVjabbAgaMjabbccaGiabbAha2jabbggaHjabbYgaSjabbwha1jabbwgaLbqaaiabbsha0jabb+gaVjabbsha0jabbggaHjabbYgaSjabcocaJiabb+gaVjabbAgaMjabbccaGiabbAha2jabbggaHjabbYgaSjabbwha1jabbwgaLjabbohaZjabgUcaRiabigdaXaaaaaa@52A7@) of the log of the product of the expression means. The y-axes correspond the probability that the control samples will have a value greater than the spike-in sample; values for the median and the MAD (median absolute deviation) were considered. The horizontal solid gray line corresponds to a probability of 12
 MathType@MTEF@5@5@+=feaafiart1ev1aaatCvAUfKttLearuWrP9MDH5MBPbIqV92AaeXatLxBI9gBaebbnrfifHhDYfgasaacH8akY=wiFfYdH8Gipec8Eeeu0xXdbba9frFj0=OqFfea0dXdd9vqai=hGuQ8kuc9pgc9s8qqaq=dirpe0xb9q8qiLsFr0=vr0=vr0dc8meaabaqaciaacaGaaeqabaqabeGadaaakeaadaWcaaqaaiabigdaXaqaaiabikdaYaaaaaa@2E9E@. The probability that, for a randomly sampled probeset, the control samples will have a value greater than the matched spike-in samples was modeled as the logit of a 4^*th*^order polynomial for rankit intensity. Solid (dashed) lines correspond to the logistic regression fits for the median (MAD). Diagnostic plots (a) and (c) indicate that, prior to re-loessing, the control and spike-in expression values were not equivalently centered and scaled for all signal intensities. Diagnostic plots (b) and (d) indicate that re-loessing the data provided control and spike-in expression values which were equivalently centered and but not equivalently scaled. Although loess correcting using only the set of true invariants can provide Stage II data which is adequately re-centered, issues pertaining to relative scale may remain and can invalidate the null distributions of commonly used two sample test statistics.

A distribution free analysis of the ten best datasets (original and re-loessed) reveals that removal of the empty nulls from the invariant set provides for Stage II datasets which are adequately centered but inadequately scaled. We (loosely) refer to the analysis as distribution free because it does not include distributional assumptions associated with a test statistic. The adequacy of the centering and scaling of the data is, of course, relative. The re-loessed data appears to be adequately centered in that the probability that randomly selecting a null probeset such that the average expression value for the control samples is larger than that for the spike-in samples is approximately one half regardless of the overall signal intensity. The re-loessed data appears to be inadequately scaled in that the probability that randomly selecting a null probeset such that the variation in the expression values for the control samples is larger than that for the spike-in samples is less than one half. Further this variation is dependent on the overall signal intensity. Figure 6 contains a panel of distribution free diagnostic plots to assess the adequacy of the centering and scaling of spike-in experiment (i.e., where true null fold changes are known) Stage II data. To evaluate relative centering, we propose modeling the probability that, for a randomly sampled probeset, the control samples will have a median expression value greater than the matched spike-in samples using the logit of a 4^*th *^order polynomial for rankit intensity. To evaluate relative scaling, we propose modeling the probability that, for a randomly sampled probeset, the control samples will have a median absolute deviation (MAD) greater than the matched spike-in samples using the logit of a function of a 4^*th *^order polynomial for rankit intensity. Given that there were only three replicates in the Golden Spike dataset we used the average of the two absolute deviations from the median value in place of the more common formulation of the MAD (which would have provided only the minimum of the two non-zero absolute deviations). The curves presented in Figure 6 correspond to the logistic regression fitted values. Note that the curves corresponding to the relative centering of the expression values (solid lines) are consistent with the biases observed in the t-statistics (depicted in Figure 4).

Table 1 contains results which indicate that the removal of the empty nulls from the invariant set provides for Stage II datasets which are adequately centered but are still inadequately scaled. For each of the 20 datasets considered, logistic models were fit as described above (i.e., the appropriate probability was modeled as the logit of a 4^*th *^order polynomial for rankit intensity) and were tested against a null model that the appropriate probability was constant with respect to rankit intensity. The deviances and asymptotic p-values (in bold) are reported. Given the possibility for cross hybridization of probesets, the assumption that the observed expression values are independent is dubious, although less tenuous than in a non-controlled experiment. The p-values, albeit approximate, indicate that the relationship between relative centering and intensity is highly significant in the original datasets and insignificant at a marginal level of 0.05 for all re-loessed datasets save 10e. The tabulated results indicate that the relationship between relative variability and intensity is highly significant for all datasets. However, the deviance values are significantly improved for several re-loessed datasets, most notably 10a-d.

**Figure 7:**
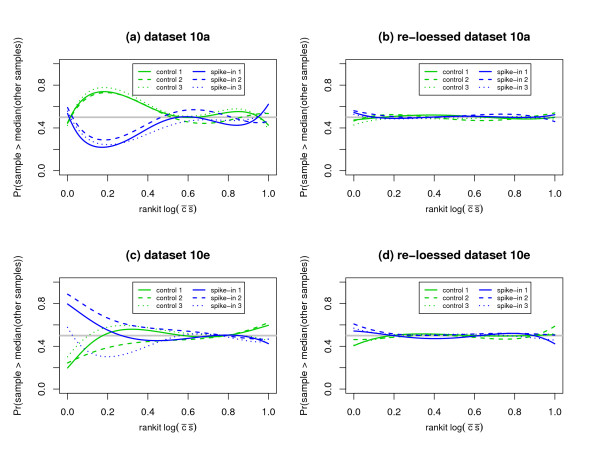
A diagnostic plot to assess, as a function of signal intensity, whether or not the underlying distributions for the expression values from each chip/sample share the same center. The x-axes correspond to the rankit (i.e., rank of valuetotal#of values+1
 MathType@MTEF@5@5@+=feaafiart1ev1aaatCvAUfKttLearuWrP9MDH5MBPbIqV92AaeXatLxBI9gBaebbnrfifHhDYfgasaacH8akY=wiFfYdH8Gipec8Eeeu0xXdbba9frFj0=OqFfea0dXdd9vqai=hGuQ8kuc9pgc9s8qqaq=dirpe0xb9q8qiLsFr0=vr0=vr0dc8meaabaqaciaacaGaaeqabaqabeGadaaakeaadaWcaaqaaiabbkhaYjabbggaHjabb6gaUjabbUgaRjabbccaGiabb+gaVjabbAgaMjabbccaGiabbAha2jabbggaHjabbYgaSjabbwha1jabbwgaLbqaaiabbsha0jabb+gaVjabbsha0jabbggaHjabbYgaSjabcocaJiabb+gaVjabbAgaMjabbccaGiabbAha2jabbggaHjabbYgaSjabbwha1jabbwgaLjabbohaZjabgUcaRiabigdaXaaaaaa@52A7@) of the log of the product of the expression means. The y-axes correspond the probability that the observed expression value for a given sample will exceed the median of the expression values for the samples not under direct examination. The horizontal solid gray line corresponds to a probability of rank of valuetotal#of values+1
 MathType@MTEF@5@5@+=feaafiart1ev1aaatCvAUfKttLearuWrP9MDH5MBPbIqV92AaeXatLxBI9gBaebbnrfifHhDYfgasaacH8akY=wiFfYdH8Gipec8Eeeu0xXdbba9frFj0=OqFfea0dXdd9vqai=hGuQ8kuc9pgc9s8qqaq=dirpe0xb9q8qiLsFr0=vr0=vr0dc8meaabaqaciaacaGaaeqabaqabeGadaaakeaadaWcaaqaaiabbkhaYjabbggaHjabb6gaUjabbUgaRjabbccaGiabb+gaVjabbAgaMjabbccaGiabbAha2jabbggaHjabbYgaSjabbwha1jabbwgaLbqaaiabbsha0jabb+gaVjabbsha0jabbggaHjabbYgaSjabcocaJiabb+gaVjabbAgaMjabbccaGiabbAha2jabbggaHjabbYgaSjabbwha1jabbwgaLjabbohaZjabgUcaRiabigdaXaaaaaa@52A7@. The probability that the sample under consideration will have an expression value greater than the median of the expression values for the samples not under direct examination, was modeled as the logit of a 4^*th *^order polynomial for rankit intensity. Colored lines correspond to the logistic regression fit values. The within subpopulation logistic model fits are remarkably consistent for dataset 10a and are less so for dataset 10e. Plots (a) and (b) suggest that problems with relative centering can not be attributed to one or two "outlying" samples. Rather, these plots support the hypothesis that the Stage I pre-processing algorithms could not adequately adjust for differences in the underlying population distributions of the expression values for the empty probesets.

A set of diagnostic plots were created to assess whether the differences in relative centering and variability could be attributed to a one or two rogue samples. Figure 7 includes an example panel of the diagnostic plots for sample datasets 10a and 10e. These plots constitute a variation on the theme of the plots presented in Figure 6. The probability that a given sample will have an expression value greater than the median of the expression values for the balance of samples was modeled as the logit of a 4^*th *^order polynomial for rankit intensity. Inspection of Figures 6(a) and 6(c) reveal that the within subpopulation (i.e., control and spike-in) logistic model fits are remarkably consistent for dataset 10a and are less so for dataset 10e. None of the plots support the hypothesis that a minority of the samples (i.e., one or two samples) are wildly inconsistent with the majority.

### Diagnostic Plots Applied to the Affymetrix SpikeInSubset Data

**Figure 8:**
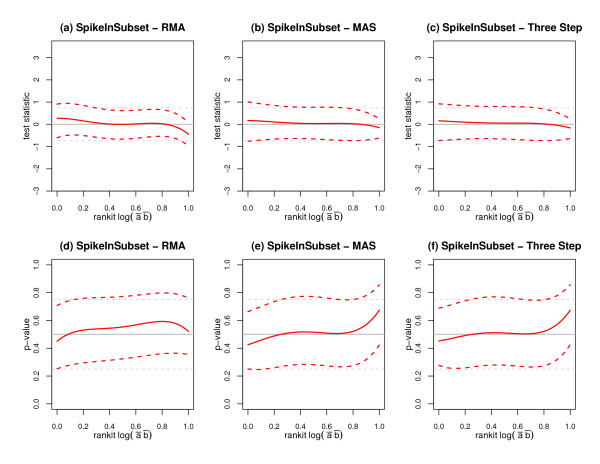
Estimates of the null t-statistic and p-value quartiles vary as a function of signal intensity for the Affymetrix SpikeInSubset dataset. The dataset was processed using Bioconductor implementations of the RMA, MAS, and three-step normalization functions. The x-axes correspond to the rankit (i.e., rank of valuetotal#of values+1
 MathType@MTEF@5@5@+=feaafiart1ev1aaatCvAUfKttLearuWrP9MDH5MBPbIqV92AaeXatLxBI9gBaebbnrfifHhDYfgasaacH8akY=wiFfYdH8Gipec8Eeeu0xXdbba9frFj0=OqFfea0dXdd9vqai=hGuQ8kuc9pgc9s8qqaq=dirpe0xb9q8qiLsFr0=vr0=vr0dc8meaabaqaciaacaGaaeqabaqabeGadaaakeaadaWcaaqaaiabbkhaYjabbggaHjabb6gaUjabbUgaRjabbccaGiabb+gaVjabbAgaMjabbccaGiabbAha2jabbggaHjabbYgaSjabbwha1jabbwgaLbqaaiabbsha0jabb+gaVjabbsha0jabbggaHjabbYgaSjabcocaJiabb+gaVjabbAgaMjabbccaGiabbAha2jabbggaHjabbYgaSjabbwha1jabbwgaLjabbohaZjabgUcaRiabigdaXaaaaaa@52A7@) of the log of the product of the expression means. The y-axes correspond to the observed t-test statistics and the observed two-sided p-values for the top and bottom rows, respectively. Solid and dashed grey lines indicate the theoretical medians and quartiles, respectively. The null p-values and t-statistics were modeled as a function of a 4^*th *^order polynomial for rankit intensity. Black and red lines correspond to the quantile regression fits for *τ *= 0.5 (solid) and *τ *= 0.25, 0.75 (dashed). The plots suggest that the null distributions may be intensity dependent, although not to the extent as was observed in the original Golden Spike datasets. Accurate quantification of the statistical significance associated with the observed intensity dependence requires an understanding of the underlying correlation structure across expression measures and remains an open research question.

**Figure 9:**
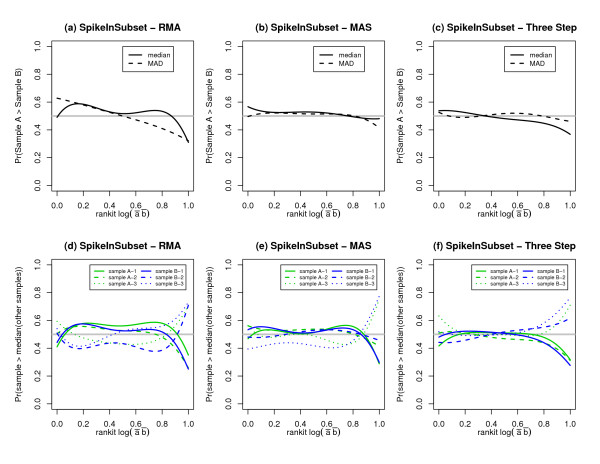
Diagnostic plots applied to the Affymetrix SpikeInSubset dataset. Axes for the top row of plots are as described in Figure 6. Axes for the bottom row of plots are as describes in Figure 7. The relative variability of the expression values appears to be intensity dependent when the data is normalized using RMA. Plots (d), (e), and (f) suggest that the intensity dependence is most notable at the extremes.

Diagnostic plots were created for the Affymetrix "SpikeInSubset" data contained in the Bioconductor [9] SpikeInSubset [10] package. The experiment was part of a larger experiment consisting of a series of transcripts spiked-in at known concentrations and arrayed in a Latin Square format. Figures 8 and 9 contain results for a six array subset (2 sets of triplicates) of the original experiment. Specifically, diagnostic plots were created for the stage II datasets created by the application of the RMA, threestep, and MAS preprocessing algorithms to the raw data.

Figure 8 suggests that the null distributions may be intensity dependent, although not to the extent as was observed in the original Golden Spike datasets. This result is expected as the Affymetrix SpikeInSubset experiment contained a smaller quantity of spiked in transcripts and did not contain anomalies of the type described in [5]. Figure 9 suggests that the intensity dependence is most notable at the extremes and that the relative variability of the expression values appears to be intensity dependent when the data is normalized using the RMA algorithm.

This analysis does not constitute a thorough investigation of the suitability of the SpikeInSubset data for validation of FDR estimation techniques. Unlike the Golden Spike data set, only a few naive "out of the box" algorithms were applied to the raw data (rather than an analysis which spans many possible combinations and settings). Figures 8 and 9 have been included to illustrate that the diagnostic plots can detect differences between (and possible defects within) the underlying putative null distributions associated with different normalization procedures. These figure also highlight the need for the development of formal methods to assess the statistical significance of such results.

## Conclusion

The Golden Spike dataset was generated to address a dearth of controlled spike-in array datasets. The original analysis of the data was presented in [1] and concluded, among other things, that common methods to control the false discovery rate had failed to control at the nominal level. Dabney and Storey determined that the failure of the FDR algorithms was not methodological, rather the distributions of the null p-values corresponding to the most common choices for the Stage II analysis (e.g., t-test, permutation t-test, and Wilcoxon Rank Sum test) were non-uniform for the datasets which were considered. Dabney and Storey concluded that the Stage II model assumptions (e.g., that the denominator of t-test is appropriate estimator of the underlying variation) associated with these tests are not met, as the non-uniform p-value distributions imply that the technical replicates generated by the experiment do not constitute adequate approximations of biological replicates. We note that their simulation parameters are provided without justification and we demonstrate that their model is inconsistent with the observed p-value distributions. In demonstrating that their analysis is flawed, we conclude that the adequacy of the technical/biological replicate approximation remains an open research question. Such a result has relevance with respect to the design of future spike-in experiments.

We suspect that the underlying null distributions are adversely effected by failures of the normalization algorithms to properly account for the abnormal feature level characteristics identified by [5]. While Irizarry et al. speculate that the Golden Spike data may not be appropriate for the comparison of methods for FDR control, we confirm that this is the case and we demonstrate the failure of the Stage I analyses to correct for systematic biases (whatever their cause may be) in the raw data matrix.

Our analysis of the Golden Spike data also reveals that the invariant set of genes used for the pre-processing steps in Choe et al. should not have included the empty null probesets. We demonstrate that removing the empty probesets from the invariant set can provide Stage II data which appears to be adequately re-centered, a result which is dependent upon artificial knowledge of the true invariant set. Unfortunately, even under these ideal conditions, issues pertaining to higher moments (e.g., relative scale) remain and these issues appear to invalidate the null distributions of commonly used two sample test statistics.

Our analysis constitutes proof of principle that the distributions of the p-values, tests statistics, and probabilities associated with the relative locations and variabilities of the expression values can vary with signal intensity. This implies that Stage I algorithms do not always adequately adjust for intensity dependent effects. If the variation of the expression values for the null genes is a consequence of the unbalanced design of the experiment, then it is reasonable to speculate the existence of biological conditions which could engender similar imbalances. For example, such imbalances could occur when comparing different tissue types, in cases of immune challenge or in certain developmental time course studies. Although it remains an open research question whether our findings apply to other datasets we note the assessment by Irizarry et al. that experiments for which normalization assumptions do not hold are becoming more common.

If the diagnostics which we have introduced prove useful for other datasets, then questions of optimality must be considered. For example, one of the diagnostic tests was based upon a 4^*th *^order polynomial logistic regression model. The order and nature of the model were chosen for computational convenience. A higher order model or a spline based approach could conceivably provide an improved diagnostic. However, the properties of the diagnostic are dependent on the unknown multivariate distribution of the feature level values for invariant genes. In order to compare statistical tests for intensity dependence of the p-values we would need to characterize the multivariate distribution of the invariant probes. This is very difficult due to the complicated correlation structure among the probes (e.g., correlations due to cross hybridization); a correlation structure that may vary from experiment to experiment. Thus the task of optimizing the diagnostics is fraught with challenges and has been relegated to future research.

## Methods

The Golden Spike dataset was generated according to the experimental design described in [1] and clarified in Figure 5 of [2]. The Golden Spike data was "re-loessed" using an invariant set which only contained the present null probesets. These calculations were performed at our request by the authors of [1] using analysis scripts which were identical to those used for the original analyses except for passages of the code relating to the identification of the invariant set. All calculations and figures presented in this manuscript were conducted using the R language and environment [11].

The Affymetrix "SpikeInSubset" data is contained in the Bioconductor [9] SpikeInSubset package [10]. The RMA [12,13], threestep, and MAS 5.0 [14] methods were applied using functions available in the "affy" [15] and "affyPLM" [16] R packages. When using the threestep procedure we chose a background subtraction using the Ideal mismatch [14], quantile normalization [17], and Tukey's Biweight [18] method for summarization. Our sample IDs are as follows: sample A-1 = 1521a99hpp_av06, sample A-2 = 1532a99hpp_av04, sample A-3 = 2353a99hpp_av08, sample B-1 = 1521b99hpp_av06, sample B-2 = 1532b99hpp_av04, sample B-3 = 2353b99hpp_av08r.

## Authors' contributions

DG developed the analyses plan, conceived of the diagnostics and authored the required R code. JM tested the R code and contributed refinements to the analysis plan and the diagnostics. DG and JM contributed equally to the development of the manuscript.

## Figures and Tables

**Table 1:** Results of logistic regression for intensity dependence.

	Median				MAD			
dataset	original		re-loess		original		re-loess	

9a	184	(**8.23e-39**)	4.37	(**0.358**)	81.5	(**8.28e-17**)	62.6	(**8.26e-13**)
9b	246	(**5.02e-52**)	3.2	(**0.525**)	48.1	(**9.06e-10**)	49.9	(**3.79e-10**)
9c	225	(**1.56e-47**)	3.41	(**0.492**)	83.4	(**3.26e-17**)	62.3	(**9.6e-13**)
9d	271	(**1.85e-57**)	4.02	(**0.403**)	71.7	(**9.93e-15**)	59	(**4.73e-12**)
9e	104	(**1.28e-21**)	7.61	(**0.107**)	24.2	(**7.38e-05**)	45.3	(**3.37e-09**)
10a	151	(**1e-31**)	6.61	(**0.158**)	82.3	(**5.69e-17**)	35.6	(**3.47e-07**)
10b	190	(**4.86e-40**)	3.19	(**0.527**)	102	(**4.63e-21**)	32.5	(**1.54e-06**)
10c	214	(**4.52e-45**)	8.12	(**0.0874**)	124	(**6.76e-26**)	47.7	(**1.11e-09**)
10d	238	(**2.1e-50**)	4.62	(**0.329**)	157	(**6.06e-33**)	39.4	(**5.63e-08**)
10e	105	(**8.49e-22**)	12	(**0.0171**)	21.9	(**0.000208**)	36.4	(**2.43e-07**)

The probability that the control samples will have a value greater than the matched spike-in samples was modeled as the logit of a function of a 4^*th *^order polynomial for rankit intensity. Values for the median and the MAD (median absolute deviation) were considered. The deviances and p-values (in bold) for the comparison of the polynomial model to a constant null model are provided and are consistent with the results presented in Figure 6. Re-loessing the data using only the fold change 1 all but eliminates the relationships between intensity and relative centering of the two sample populations. However, the relationships between intensity and relative variability of the expression values remain, although they are greatly diminished.
